# Blood Lead Levels in Children Aged 1–5 Years — United States, 1999–2010

**Published:** 2013-04-05

**Authors:** William Wheeler, Mary Jean Brown

**Affiliations:** Div of Nutrition, Physical Activity, and Obesity, National Center for Chronic Disease Prevention and Health Promotion; Div of Emergency and Environmental Health Svcs, National Center for Environmental Health, CDC

The adverse health effects of lead exposure in children are well described and include intellectual and behavioral deficits, making lead exposure an important public health problem (1). No safe blood lead level (BLL) in children has been identified. To estimate the number of children aged 1–5 years in the United States at risk for adverse health effects from lead exposure and to assess the impact of prevention efforts, CDC analyzed data from the National Health and Nutrition Examination Survey (NHANES) from the periods 1999–2002 to 2007–2010. This report summarizes the results of that analysis, which indicated that the percentage of children aged 1–5 years with BLLs at or above the upper reference interval value of 5 *μ*g/dL calculated using the 2007–2010 NHANES cycle was 2.6%. Thus, an estimated 535,000 U.S. children aged 1–5 years had BLLs ≥5 *μ*g/dL based on the U.S. Census Bureau 2010 count of the number of children in this age group. Despite progress in reducing BLLs among children in this age group overall, differences between the mean BLLs of different racial/ethnic and income groups persist, and work remains to be done to reach the *Healthy People 2020* objective of reducing mean BLLs for all children in the United States (EH-8.2) (2).

In 1991, CDC defined BLLs ≥10 *μ*g/dL as the “level of concern” for children aged 1–5 years (3). However, in May 2012, CDC accepted the recommendations of its Advisory Committee on Childhood Lead Poisoning Prevention (ACCLPP) that the term “level of concern” be replaced with an upper reference interval value defined as the 97.5th percentile of BLLs in U.S. children aged 1–5 years from two consecutive cycles of NHANES (4). CDC conducts NHANES, a continuous, cross-sectional, representative survey of the noninstitutionalized U.S. civilian population, using a complex, multistage probability design. Since the mid-1970s, when NHANES first began measuring blood lead levels, the survey has become the basis for monitoring changes in BLLs in the United States. Beginning in 1999, NHANES became a continuous survey, with roughly 10,000 NHANES participants interviewed and examined during each 2-year cycle. Approximately 1,240 children aged 1–5 years are examined every cycle, and a blood specimen is drawn from approximately 850 (69%) of them. In NHANES, BLL is measured using inductively coupled plasma mass spectrometry in the elemental analysis laboratory at CDC (5). The current upper reference interval value of the 97.5th percentile of the distribution of the combined 2007–2008 and 2009–2010 cycles of NHANES was calculated as 5 *μ*g/dL.

For this analysis, a BLL ≥5 *μ*g/dL is defined as a high BLL. The geometric mean (GM) BLLs for children aged 1–5 years and 95% confidence intervals (CIs) also were calculated. Data are presented in 4-year aggregates from the 1999–2002, 2003–2006, and 2007–2010 NHANES cycles. Significant differences in GM between categories in selected characteristics were tested using pairwise *t*-tests. Values below the BLL limit of detection were replaced with the limit of detection divided by the square root of 2, and all data analyses included sample weights to account for unequal probabilities of selection, oversampling, and survey nonresponse (6).

This analysis was focused on demographic categories with long-standing disparities in risk for high BLLs between groups: age, sex, race/ethnicity, age of housing, poverty income ratio (PIR), and Medicaid enrollment status. Race/ethnicity was categorized as non-Hispanic white, non-Hispanic black, Mexican American, and “other.” Although children whose race/ethnicity was categorized as “other” were included in overall estimates, they were excluded from estimates stratified by race/ethnicity because of small numbers. PIR was calculated by dividing the total annual family income by the federal poverty threshold specific to family size, year, and state of residence. PIR was categorized as either <1.3 or ≥1.3 times the poverty level.

In bivariate analyses, the CI for the 2007–2010 NHANES estimates of the percentage of non-Hispanic black children (3.3%–8.4%) and non-Hispanic white children (0.7%–5.2%) with BLLs ≥5 *μ*g/dL overlap (Table 1). However, disparities in the GM BLL by factors such as race/ethnicity and income level, which have been important historically, persist. The difference between the GM BLL of non-Hispanic black children (1.8 *μ*g/dL [CI = 1.6–1.9]) GM BLL compared with either non-Hispanic white (1.3 *μ*g/dL [CI = 1.1–1.4]) or Mexican American (1.3 *μ*g/dL [CI = 1.2–1.4]) children remains significant (p<0.01) (Table 2). The difference in GM BLL among children belonging to families with a PIR <1.3 compared with families with a PIR ≥1.3 also is significant (1.6 *μ*g/dL versus 1.2 *μ*g/dL, respectively [p<0.01]), as is the difference in GM BLL by age group and Medicaid enrollment status (Table 2).

## Editorial Note

Substantial progress has been made over the past four decades in reducing the number of children with elevated BLLs. Data from the 1976–1980 cycle of NHANES indicated that an estimated 88% of children aged 1–5 years had BLLs ≥10 *μ*g/dL (7). Since then, the percentage has fallen sharply, to 4.4% during 1991–1994 (NHANES III) (8), to 1.6% during 1999–2002 (9), and to 0.8% during 2007–2010. National estimates of the GM BLL for children aged 1–5 years declined significantly over time, from a 1976–1980 estimated GM BLL of 15 *μ*g/dL (CI = 14.2–15.8) to a 1988–1991 estimated GM BLL 3.6 *μ*g/dL (CI = 3.3–4.0), and this trend continues. During 1999–2002, the GM BLL was 1.9 *μ*g/dL (CI = 1.8–2.1), compared with the 2007–2010 estimated GM BLL of 1.3 *μ*g/dL (CI = 1.3–1.4).*

What is already known on this topic?

Elevated blood lead levels (BLLs) in children cause learning and behavioral deficits. No threshold for these effects has been identified. In January 2012, CDC’s Advisory Committee on Childhood Lead Poisoning recommended that BLLs in children be kept below 5 *μ*g/dL.

What is added by this report?

The percentage of children aged 1–5 years with BLLs ≥5 *μ*g/dL from the 2007–2010 National Health and Nutritional Examination Survey cycle was 2.6%, indicating an estimated 535,000 U.S. children aged 1–5 years with BLLs ≥5 *μ*g/dL. Despite progress in reducing BLLs among children in this age group overall, long-standing disparities persist. The geometric mean BLLs (GM BLLs) among younger children, those belonging to poor families, and those enrolled in Medicaid were significantly higher compared with their older, more affluent counterparts, while the GM BLL for non-Hispanic black children was significantly higher compared with either non-Hispanic white or Mexican American children.

What are the implications for public health practice?

The greatest reductions in the proportion of children with elevated BLLs have been made over the past four decades in those racial/ethnic and income groups that had the highest BLLs. Persistent differences between the mean BLLs of different racial/ethnic and income groups can be traced to differences in housing quality, environmental conditions, nutrition, and other factors. Resources should be targeted to areas and communities where children are most at risk to achieve the *Healthy People 2020* objective of reducing mean BLLs for all children in the United States (EH 8.2).

The greatest reductions have occurred among children in racial/ethnic and income groups that historically were most likely to have BLLs ≥10 *μ*g/dL. These reductions reflect the impact of strategies coordinated and implemented at national, state, and local levels. They include elimination of lead in vehicle emissions, elimination of lead paint hazards in housing, reduction in lead concentrations in air, water, and consumer products marketed to children, and identification and increased screening of populations at high risk (3). However, the small numbers of NHANES participants with BLLs ≥10 *μ*g/dL means that national estimates of the prevalence of BLLs this high are unstable, and year-to-year changes in prevalence are difficult to interpret. In the 2007–2008 and 2009–2010 NHANES cycles, nine and six survey participants, respectively, aged 1–5 years had BLLs ≥10 *μ*g/dL.

Childhood exposure to lead can have lifelong consequences. The significant differences between the GM BLLs by race/ethnicity and income indicate a persistent disparity. In January 2012, ACCLPP observed that these disparities can be traced to differences in housing quality, environmental conditions, nutrition, and other factors designed to control or eliminate lead exposure (4).

CDC concurred with ACCLPP that primary prevention (i.e., ensuring that all homes are lead-safe and do not contribute to childhood lead exposure) is the only practical approach to preventing elevated BLLs in children (10). Prevention requires reducing environmental exposures from soil, dust, paint, and water, before children are exposed to these hazards. Efforts to increase awareness of lead hazards and nutritional interventions to increase iron and calcium, which can reduce lead absorption, are other key components of a successful prevention policy (4). Given the continued disparity in BLLs, resources should be targeted to those areas where children are most at risk. NHANES provides useful data for measuring progress towards eliminating high BLLs and ensuring that resources are targeted toward the most vulnerable children.

## Figures and Tables

**TABLE 1:** Number sampled and estimated percentage of children aged 1–5 years with blood lead levels ≥5 *μ*g/dL, by selected characteristics — United States, National Health and Nutrition Examination Survey, 1999–2002, 2003–2006, and 2007–2010

	1999–2002			2003–2006			2007–2010		
			
Characteristic	No.	%	(95% CI)	No.	%	(95% CI)	No.	%	(95% CI)
**Total**	**1,621**	**8.6**	**(6.3–11.3)**	**1,879**	**4.1**	**(2.8–5.7)**	**1,653**	**2.6**	**(1.6–4.0)**
**Sex**									
Boy	851	9.1	(5.9–12.9)	951	3.9	(2.4–5.8)	872	2.5	(1.3–4.1)
Girl	770	8.2	(6.0–10.6)	928	4.3	(2.9–5.9)	781	2.8	(1.6–4.2)
**Age group (yrs)**									
1–2	779	12.2	(9.1–15.6)	919	5.7	(4.3–7.2)	793	3.1	(2.1–4.4)
3–5	842	6.4	(3.8–9.6)	960	3.0	(1.5–5.1)	860	2.3	(0.9–4.4)*
**Race/Ethnicity**									
Black, non-Hispanic	454	18.5	(13.7–23.8)	546	12.1	(6.5–19.2)	338	5.6	(3.3–8.4)
Mexican American	541	7.4	(4.7–10.6)	611	2.6	(1.1–4.6)	490	1.9	(0.7–3.7)*
White, non-Hispanic	465	7.1	(3.7–11.5)	540	2.3	(1.4–3.2)	536	2.4	(0.7–5.2)*
**Poverty income ratio**									
<1.3	817	12.9	(9.5–16.7)	941	8.1	(5.2–11.6)	868	4.4	(3.0–6.2)
≥1.3	677	4.5	(2.6–6.7)	852	1.6	(0.7–2.9)*	642	1.2	(0.1–3.7)*
**Age of housing**									
Pre-1950	208	18.4	(13.1–24.4)	242	8.8	(5.3–13.2)	264	5.3	(1.1–12.6)*
1950–1977	341	5.3	(2.9–8.4)	413	2.2	(0.8–4.3)*	343	1.3	(0.6–2.4)*
1978 or later	470	2.1	(0.9–3.7)*	528	1.4	(0.6–2.4)*	503	0.4	(0.1–1.0)*
Refused/Don’t know	602	15.0	(10.7–19.9)	696	7.5	(3.6–12.6)	543	5.1	(3.3–7.4)
**Medicaid enrollment status**									
Yes	592	15.1	(11.5–19.1)	740	7.1	(4.5–10.1)	633	4.3	(2.8–6.1)
No	998	6.0	(3.9–8.5)	1,127	2.9	(1.9–4.0)	1,019	2.0	(0.9–3.4)*

**Abbreviation:** CI = confidence interval.

* Estimate is statistically unreliable (relative standard error is ≥30).

**TABLE 2:** Number sampled and estimated geometric mean blood lead levels (GM BLLs) of children aged 1–5 years, by selected characteristics — United States, National Health and Nutrition Examination Survey, 1999–2002, 2003–2006, and 2007–2010

	1999–2002			2003–2006			2007–2010		
			
Characteristic	No.	GM BLL (*μ*g/dL)	(95% CI)	No.	GM BLL (*μ*g/dL)	(95% CI)	No.	GM BLL (*μ*g/dL)	(95% CI)
**Total**	**1,621**	**1.9**	**(1.8–2.1)**	**1,879**	**1.6**	**(1.5–1.7)**	**1,653**	**1.3**	**(1.3–1.4)** *
**Sex**									
Boy	851	1.9	(1.8–2.1)	951	1.6	(1.5–1.7)	872	1.3	(1.3–1.4)*
Girl	770	1.9	(1.8–2.1)	928	1.6	(1.5–1.7)	781	1.3	(1.2–1.4)
**Age group (yrs)**									
1–2	779	2.2	(2.0–2.4)	919	1.8	(1.7–1.9)	793	1.5	(1.4–1.6)
3–5	842	1.8	(1.6–2.0)	960	1.5	(1.4–1.6)	860	1.2	(1.2–1.3)*
**Race/Ethnicity**									
Black, non-Hispanic	454	2.8	(2.5–3.1)	546	2.4	(2.1–2.8)	338	1.8	(1.6–1.9)
Mexican American	541	1.9	(1.8–2.0)	611	1.6	(1.5–1.7)	490	1.3	(1.2–1.4)
White, non-Hispanic	465	1.8	(1.6–2.0)	540	1.5	(1.4–1.5)*	536	1.3	(1.1–1.4)
**Poverty income ratio**									
<1.3	817	2.4	(2.2–2.7)	941	2.0	(1.8–2.2)	868	1.6	(1.5–1.7)
≥1.3	677	1.6	(1.4–1.7)	852	1.4	(1.3–1.5)	642	1.2	(1.1–1.3)
**Age of housing**									
Pre-1950	208	2.7	(2.4–3.1)	242	2.1	(1.8–2.3)	264	1.6	(1.4–1.9)
1950–1977	341	1.8	(1.7–2.0)	413	1.5	(1.4–1.7)	343	1.3	(1.2–1.5)
1978 or later	470	1.5	(1.3–1.6)	528	1.3	(1.2–1.4)	503	1.1	(1.0–1.2)
Refused/Don’t know	602	2.5	(2.2–2.7)	696	2.0	(1.8–2.3)	543	1.6	(1.5–1.7)
**Medicaid enrollment status**									
Yes	592	2.6	(2.4–2.8)	740	2.0	(1.8–2.2)	633	1.6	(1.5–1.7)
No	998	1.7	(1.6–1.9)	1,127	1.5	(1.4–1.6)	1,019	1.2	(1.2–1.3)*

**Abbreviation:** CI = confidence interval.

* Where CIs are equal to the point estimate, this is because of rounding.

## Footnotes

* Where CIs are equal to the point estimate, this is because of rounding.
